# Tricarbonyl­bis­(1,3-diisopropyl-4,5-dimethyl­imidazol-2-yl­idene)iron

**DOI:** 10.1107/S1600536812048179

**Published:** 2012-11-28

**Authors:** Aziza Ahmida, Ulrich Flörke, Gerald Henkel

**Affiliations:** a Department Chemie, Fakultät für Naturwissenschaften, Universität Paderborn, Warburgerstrasse 100, D-33098 Paderborn, Germany

## Abstract

In the title compound, [Fe(C_11_H_20_N_2_)_2_(CO)_3_], the Fe atom shows a distorted trigonal–bipyramidal geometry with three carbonyl and two carbene ligands. The latter have a *cis* arrangement, with a C—Fe—C angle of 90.60 (7)°. The Fe atom lies 0.007 (1) Å above the basal plane defined by two carbonyl and one carbene C atoms. The mol­ecular structure is closely related to that of the isomolecular but not isotypic Ru complex with an identical *cis* arrangement, so, in general, bond geometries lie in expected ranges. In the crystal, C—H⋯O hydrogen bonds link the mol­ecules into infinite zigzag chains extending along [010].

## Related literature

For structures of related *cis* complexes, see: Ellul *et al.* (2008 ▶). For Co and Ru complexes with a *trans* configuration, see: van Rendsburg *et al.* (2007 ▶); Chantler *et al.* (2008 ▶); Ellul *et al.* (2008 ▶).
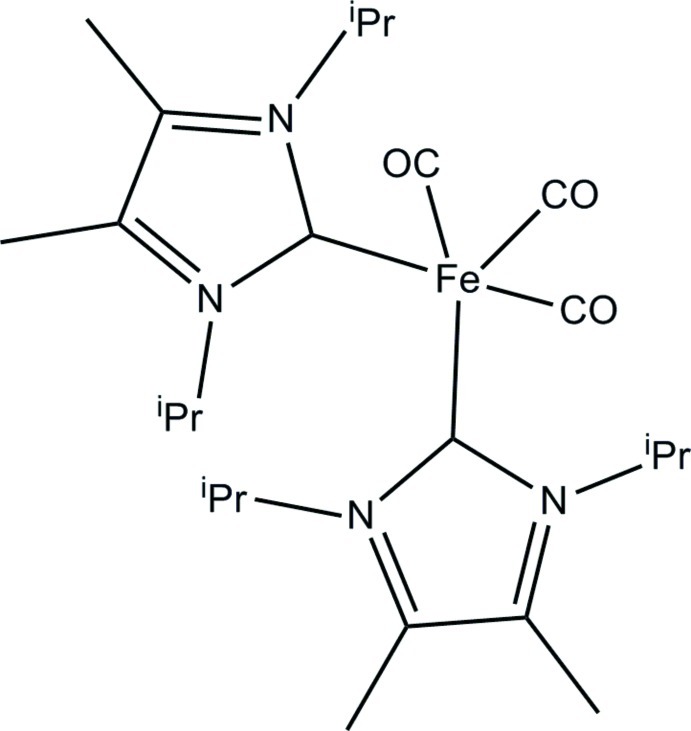


## Experimental

### 

#### Crystal data

[Fe(C_11_H_20_N_2_)_2_(CO)_3_]
*M* *_r_* = 500.46
Orthorhombic, 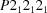

*a* = 11.5913 (5) Å
*b* = 12.7572 (5) Å
*c* = 17.7970 (7) Å
*V* = 2631.69 (19) Å^3^
*Z* = 4
Mo *K*α radiation
μ = 0.61 mm^−1^
*T* = 120 K
0.38 × 0.32 × 0.29 mm

#### Data collection

Bruker SMART CCD area-detector diffractometer
Absorption correction: multi-scan (*SADABS*; Sheldrick, 2004 ▶) *T*_min_ = 0.803, *T*_max_ = 0.844
26635 measured reflections
6362 independent reflections
5983 reflections with *I* > 2σ(*I*)
*R*_int_ = 0.035

#### Refinement

*R*[*F* ^2^ > 2σ(*F* ^2^)] = 0.033
*wR*(*F* ^2^) = 0.082
*S* = 1.04
6362 reflections
298 parameters
H-atom parameters constrained
Δρ_max_ = 0.42 e Å^−3^
Δρ_min_ = −0.23 e Å^−3^
Absolute structure: Flack (1983 ▶), 2789 Friedel pairs
Flack parameter: 0.022 (11)

### 

Data collection: *SMART* (Bruker, 2002 ▶); cell refinement: *SAINT* (Bruker, 2002 ▶); data reduction: *SAINT*; program(s) used to solve structure: *SHELXTL* (Sheldrick, 2008 ▶); program(s) used to refine structure: *SHELXTL*; molecular graphics: *SHELXTL*; software used to prepare material for publication: *SHELXTL* and local programs.

## Supplementary Material

Crystal structure: contains datablock(s) I, global. DOI: 10.1107/S1600536812048179/bt6867sup1.cif

Structure factors: contains datablock(s) I. DOI: 10.1107/S1600536812048179/bt6867Isup2.hkl

Additional supplementary materials:  crystallographic information; 3D view; checkCIF report

## Figures and Tables

**Table 1:** Hydrogen-bond geometry (Å, °)

*D*—H⋯*A*	*D*—H	H⋯*A*	*D*⋯*A*	*D*—H⋯*A*
C25—H25*B*⋯O2^i^	0.98	2.42	3.154 (3)	131

Symmetry code: (i) 
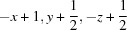
.

**Fig. 1.**
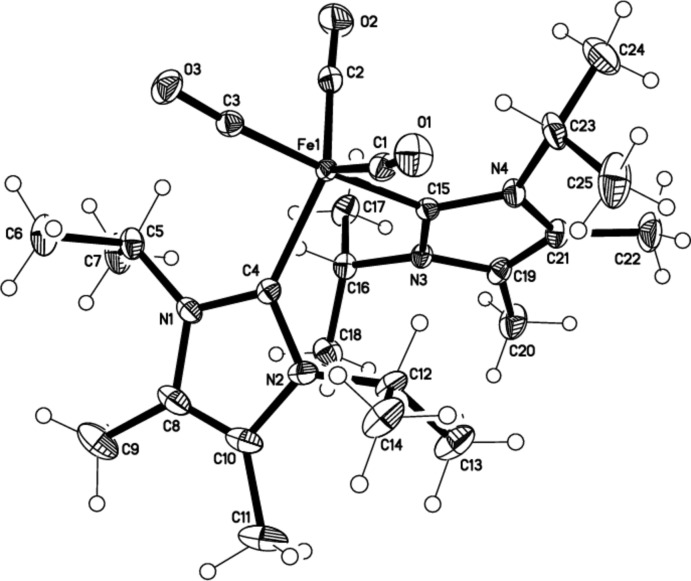
Molecular structure of the title compound. Displacement ellipsoids are drawn at the 50% probability level.

**Fig. 2.**
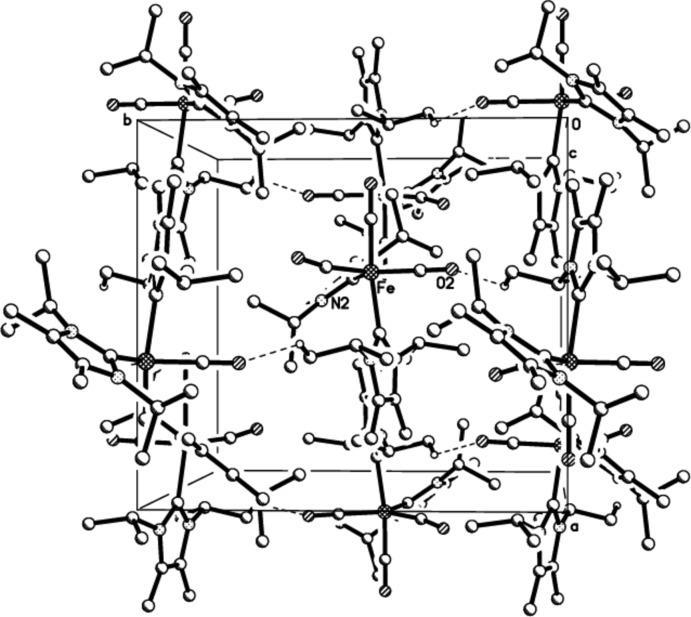
Crystal packing viewed along [001] with hydrogen bonding pattern indicated as dashed lines. H-atoms not involved in bonding are omitted.

## supplementary crystallographic information

### Experimental

To a solution of 1,3-diisopropyl-4,5-dimethylimidazol-2-thione (850 mg, 4 mmol) in 30 ml THF was added K (420 mg, 1 mmol + 10% excess) and the mixture was refluxed under inert atmosphere for 4 h. After filtration and removal of K_2_S, Fe_2_(CO)_9_ (360 mg, 1 mmol) was added to the filtrate. The reaction mixture was stirred over night at room temperature. After removal of the solvent and subsequent drying in vacuum the residue was cyrstallized by diffusion of hexane into a concentrated toluene solution to give single-crystal of the title complex.

### Refinement

All Hydrogen atom positions were clearly derived from difference maps, then refined at calculated positions riding on the parent atoms with C—H 0.98 - 1.00 Å and isotropic displacement parameters *U*_iso_(H) = 1.2U(C_eq_) or 1.5U(–CH_3_). All CH_3_ hydrogen atoms were allowed to rotate but not to tip.

### Crystal data

**Table:**

[Fe(C_11_H_20_N_2_)_2_(CO)_3_]	*F*(000) = 1072
*M**_r_* = 500.46	*D*_x_ = 1.263 Mg m^−^^3^
Orthorhombic, *P*2_1_2_1_2_1_	Mo *K*α radiation, λ = 0.71073 Å
Hall symbol: P 2ac 2ab	Cell parameters from 7772 reflections
*a* = 11.5913 (5) Å	θ = 2.4–27.7°
*b* = 12.7572 (5) Å	µ = 0.61 mm^−^^1^
*c* = 17.7970 (7) Å	*T* = 120 K
*V* = 2631.69 (19) Å^3^	Prism, yellow
*Z* = 4	0.38 × 0.32 × 0.29 mm

### Data collection

**Table:**

Bruker SMART CCD area-detector diffractometer	6362 independent reflections
Radiation source: sealed tube	5983 reflections with *I* > 2σ(*I*)
Graphite monochromator	*R*_int_ = 0.035
φ and ω scans	θ_max_ = 28.1°, θ_min_ = 2.0°
Absorption correction: multi-scan (*SADABS*; Sheldrick, 2004)	*h* = −15→15
*T*_min_ = 0.803, *T*_max_ = 0.844	*k* = −16→16
26635 measured reflections	*l* = −23→23

### Refinement

**Table:**

Refinement on *F*^2^	Secondary atom site location: difference Fourier map
Least-squares matrix: full	Hydrogen site location: difference Fourier map
*R*[*F*^2^ > 2σ(*F*^2^)] = 0.033	H-atom parameters constrained
*wR*(*F*^2^) = 0.082	*w* = 1/[σ^2^(*F*_o_^2^) + (0.0461*P*)^2^ + 0.1759*P*] where *P* = (*F*_o_^2^ + 2*F*_c_^2^)/3
*S* = 1.04	(Δ/σ)_max_ = 0.002
6362 reflections	Δρ_max_ = 0.42 e Å^−^^3^
298 parameters	Δρ_min_ = −0.23 e Å^−^^3^
0 restraints	Absolute structure: Flack (1983), 2789 Friedel pairs
Primary atom site location: structure-invariant direct methods	Flack parameter: 0.022 (11)

### Special details

**Table:**

Geometry. All e.s.d.'s (except the e.s.d. in the dihedral angle between two l.s. planes) are estimated using the full covariance matrix. The cell e.s.d.'s are taken into account individually in the estimation of e.s.d.'s in distances, angles and torsion angles; correlations between e.s.d.'s in cell parameters are only used when they are defined by crystal symmetry. An approximate (isotropic) treatment of cell e.s.d.'s is used for estimating e.s.d.'s involving l.s. planes.
Refinement. Refinement of *F*^2^ against ALL reflections. The weighted *R*-factor *wR* and goodness of fit *S* are based on *F*^2^, conventional *R*-factors *R* are based on *F*, with *F* set to zero for negative *F*^2^. The threshold expression of *F*^2^ > σ(*F*^2^) is used only for calculating *R*-factors(gt) *etc*. and is not relevant to the choice of reflections for refinement. *R*-factors based on *F*^2^ are statistically about twice as large as those based on *F*, and *R*- factors based on ALL data will be even larger.

### Fractional atomic coordinates and isotropic or equivalent isotropic displacement parameters (Å^2^)

**Table:**

	*x*	*y*	*z*	*U*_iso_*/*U*_eq_	
Fe1	0.378885 (18)	0.495342 (17)	0.430547 (11)	0.01450 (6)	
O1	0.34212 (13)	0.66917 (12)	0.32606 (8)	0.0353 (4)	
O2	0.37267 (15)	0.28447 (11)	0.36881 (9)	0.0422 (4)	
O3	0.12817 (10)	0.49640 (11)	0.43365 (7)	0.0306 (3)	
N1	0.34487 (13)	0.52000 (12)	0.60226 (8)	0.0216 (3)	
N2	0.45810 (13)	0.63918 (11)	0.55801 (9)	0.0219 (3)	
N3	0.62444 (13)	0.43646 (11)	0.47979 (7)	0.0181 (3)	
N4	0.62449 (12)	0.49072 (12)	0.36536 (8)	0.0216 (3)	
C1	0.36558 (16)	0.60380 (14)	0.36833 (10)	0.0213 (3)	
C2	0.37532 (17)	0.36667 (14)	0.39603 (10)	0.0229 (4)	
C3	0.22766 (14)	0.49610 (14)	0.43548 (9)	0.0199 (3)	
C4	0.39480 (14)	0.55369 (13)	0.53668 (9)	0.0171 (3)	
C5	0.26875 (16)	0.42794 (16)	0.60580 (11)	0.0251 (4)	
H5A	0.2674	0.3977	0.5540	0.030*	
C6	0.14431 (18)	0.45811 (19)	0.62406 (13)	0.0364 (5)	
H6A	0.1193	0.5145	0.5904	0.055*	
H6B	0.1396	0.4822	0.6762	0.055*	
H6C	0.0942	0.3970	0.6173	0.055*	
C7	0.3120 (2)	0.34124 (18)	0.65709 (13)	0.0370 (5)	
H7A	0.3918	0.3238	0.6439	0.056*	
H7B	0.2633	0.2790	0.6510	0.056*	
H7C	0.3088	0.3650	0.7094	0.056*	
C8	0.3765 (2)	0.58435 (17)	0.66267 (10)	0.0324 (4)	
C9	0.3397 (3)	0.5679 (3)	0.74274 (13)	0.0557 (8)	
H9A	0.3625	0.6286	0.7730	0.084*	
H9B	0.3768	0.5047	0.7627	0.084*	
H9C	0.2557	0.5595	0.7448	0.084*	
C10	0.44711 (19)	0.65845 (16)	0.63532 (11)	0.0311 (5)	
C11	0.5096 (3)	0.74236 (19)	0.67739 (14)	0.0511 (7)	
H11A	0.4736	0.7521	0.7267	0.077*	
H11B	0.5058	0.8081	0.6490	0.077*	
H11C	0.5905	0.7219	0.6840	0.077*	
C12	0.52171 (16)	0.70393 (14)	0.50370 (12)	0.0255 (4)	
H12A	0.5184	0.6652	0.4549	0.031*	
C13	0.64936 (17)	0.71613 (17)	0.52206 (15)	0.0382 (5)	
H13A	0.6839	0.6468	0.5295	0.057*	
H13B	0.6580	0.7577	0.5680	0.057*	
H13C	0.6884	0.7518	0.4804	0.057*	
C14	0.46139 (18)	0.80827 (16)	0.48996 (14)	0.0370 (5)	
H14A	0.3798	0.7957	0.4786	0.055*	
H14B	0.4979	0.8441	0.4474	0.055*	
H14C	0.4677	0.8521	0.5350	0.055*	
C15	0.55252 (14)	0.47282 (12)	0.42458 (10)	0.0170 (3)	
C16	0.58137 (15)	0.38014 (14)	0.54622 (10)	0.0199 (4)	
H16A	0.4960	0.3912	0.5475	0.024*	
C17	0.60057 (17)	0.26248 (14)	0.53661 (12)	0.0293 (4)	
H17A	0.5680	0.2397	0.4885	0.044*	
H17B	0.5626	0.2248	0.5777	0.044*	
H17C	0.6835	0.2475	0.5375	0.044*	
C18	0.62846 (19)	0.42159 (16)	0.62020 (10)	0.0291 (4)	
H18A	0.6133	0.4970	0.6238	0.044*	
H18B	0.7118	0.4092	0.6225	0.044*	
H18C	0.5907	0.3853	0.6620	0.044*	
C19	0.73965 (15)	0.43699 (15)	0.45598 (11)	0.0226 (4)	
C20	0.84120 (17)	0.41219 (19)	0.50418 (13)	0.0359 (5)	
H20A	0.9120	0.4184	0.4744	0.054*	
H20B	0.8342	0.3405	0.5234	0.054*	
H20C	0.8442	0.4614	0.5464	0.054*	
C21	0.73951 (15)	0.47107 (14)	0.38450 (10)	0.0238 (4)	
C22	0.84160 (17)	0.4855 (2)	0.33430 (13)	0.0413 (6)	
H22A	0.9121	0.4874	0.3647	0.062*	
H22B	0.8338	0.5515	0.3066	0.062*	
H22C	0.8459	0.4270	0.2987	0.062*	
C23	0.58288 (17)	0.51908 (17)	0.28977 (10)	0.0306 (5)	
H23A	0.4967	0.5168	0.2921	0.037*	
C24	0.6189 (2)	0.4380 (3)	0.23116 (12)	0.0501 (7)	
H24A	0.5947	0.3681	0.2477	0.075*	
H24B	0.7029	0.4394	0.2252	0.075*	
H24C	0.5822	0.4545	0.1830	0.075*	
C25	0.6150 (2)	0.6308 (2)	0.26977 (15)	0.0508 (7)	
H25A	0.5880	0.6782	0.3094	0.076*	
H25B	0.5786	0.6500	0.2220	0.076*	
H25C	0.6990	0.6365	0.2650	0.076*	

### Atomic displacement parameters (Å^2^)

**Table:**

	*U*^11^	*U*^22^	*U*^33^	*U*^12^	*U*^13^	*U*^23^
Fe1	0.01323 (11)	0.01603 (11)	0.01422 (10)	−0.00051 (10)	−0.00024 (8)	0.00020 (10)
O1	0.0401 (9)	0.0333 (8)	0.0325 (8)	0.0048 (7)	−0.0021 (6)	0.0136 (6)
O2	0.0557 (10)	0.0268 (7)	0.0442 (8)	−0.0075 (8)	0.0135 (9)	−0.0155 (6)
O3	0.0164 (6)	0.0346 (7)	0.0407 (7)	0.0008 (7)	−0.0010 (5)	−0.0029 (7)
N1	0.0227 (7)	0.0253 (8)	0.0167 (7)	0.0028 (6)	0.0016 (5)	−0.0029 (6)
N2	0.0211 (7)	0.0203 (7)	0.0243 (8)	−0.0004 (6)	−0.0042 (6)	−0.0048 (6)
N3	0.0149 (7)	0.0202 (7)	0.0194 (7)	0.0003 (6)	0.0004 (6)	0.0015 (5)
N4	0.0177 (6)	0.0279 (7)	0.0191 (6)	0.0005 (8)	0.0024 (5)	0.0027 (6)
C1	0.0201 (9)	0.0236 (9)	0.0202 (8)	−0.0013 (7)	−0.0023 (7)	0.0023 (7)
C2	0.0218 (9)	0.0249 (9)	0.0220 (8)	−0.0010 (8)	0.0038 (8)	−0.0013 (7)
C3	0.0209 (7)	0.0179 (7)	0.0208 (7)	0.0006 (7)	−0.0004 (6)	−0.0014 (9)
C4	0.0137 (8)	0.0172 (8)	0.0203 (8)	0.0031 (6)	−0.0014 (6)	0.0005 (6)
C5	0.0243 (9)	0.0293 (10)	0.0218 (9)	0.0005 (8)	0.0045 (7)	0.0056 (8)
C6	0.0262 (11)	0.0460 (13)	0.0370 (11)	0.0031 (9)	0.0090 (9)	0.0084 (10)
C7	0.0353 (11)	0.0424 (13)	0.0333 (11)	0.0047 (10)	0.0089 (9)	0.0162 (9)
C8	0.0376 (11)	0.0395 (11)	0.0201 (9)	0.0073 (10)	−0.0003 (9)	−0.0089 (8)
C9	0.0736 (19)	0.0734 (19)	0.0201 (10)	−0.0006 (15)	0.0049 (11)	−0.0119 (12)
C10	0.0366 (11)	0.0313 (11)	0.0254 (10)	0.0060 (9)	−0.0067 (9)	−0.0118 (8)
C11	0.0654 (18)	0.0458 (14)	0.0423 (13)	−0.0033 (13)	−0.0141 (13)	−0.0228 (12)
C12	0.0206 (9)	0.0199 (9)	0.0359 (10)	−0.0048 (7)	−0.0035 (8)	−0.0017 (8)
C13	0.0229 (10)	0.0269 (10)	0.0649 (16)	−0.0025 (8)	−0.0079 (10)	−0.0019 (10)
C14	0.0249 (10)	0.0243 (10)	0.0618 (15)	−0.0021 (8)	−0.0080 (10)	0.0063 (10)
C15	0.0180 (8)	0.0160 (8)	0.0170 (7)	−0.0007 (6)	0.0002 (6)	−0.0007 (6)
C16	0.0186 (8)	0.0219 (9)	0.0193 (8)	0.0027 (7)	−0.0011 (7)	0.0059 (7)
C17	0.0264 (10)	0.0213 (9)	0.0402 (11)	0.0005 (7)	0.0010 (9)	0.0066 (8)
C18	0.0313 (10)	0.0353 (10)	0.0206 (9)	0.0035 (9)	−0.0040 (8)	0.0029 (7)
C19	0.0155 (8)	0.0225 (9)	0.0297 (9)	0.0003 (7)	0.0010 (7)	0.0002 (7)
C20	0.0183 (9)	0.0495 (13)	0.0399 (12)	0.0017 (9)	−0.0019 (8)	0.0084 (10)
C21	0.0168 (8)	0.0278 (10)	0.0267 (9)	0.0004 (7)	0.0039 (7)	0.0005 (7)
C22	0.0213 (9)	0.0639 (17)	0.0387 (11)	−0.0045 (10)	0.0093 (8)	0.0062 (12)
C23	0.0256 (9)	0.0482 (13)	0.0179 (8)	0.0050 (9)	0.0050 (7)	0.0080 (8)
C24	0.0374 (13)	0.088 (2)	0.0252 (11)	0.0085 (15)	0.0011 (11)	−0.0116 (12)
C25	0.0413 (13)	0.0592 (15)	0.0518 (14)	0.0049 (13)	0.0092 (12)	0.0319 (12)

### Geometric parameters (Å, º)

**Table:**

Fe1—C2	1.7531 (18)	C11—H11C	0.9800
Fe1—C3	1.7551 (16)	C12—C13	1.523 (3)
Fe1—C1	1.7788 (17)	C12—C14	1.523 (3)
Fe1—C15	2.0358 (16)	C12—H12A	1.0000
Fe1—C4	2.0386 (17)	C13—H13A	0.9800
O1—C1	1.156 (2)	C13—H13B	0.9800
O2—C2	1.156 (2)	C13—H13C	0.9800
O3—C3	1.154 (2)	C14—H14A	0.9800
N1—C4	1.372 (2)	C14—H14B	0.9800
N1—C8	1.402 (2)	C14—H14C	0.9800
N1—C5	1.470 (2)	C16—C18	1.520 (3)
N2—C4	1.368 (2)	C16—C17	1.527 (2)
N2—C10	1.403 (2)	C16—H16A	1.0000
N2—C12	1.470 (2)	C17—H17A	0.9800
N3—C15	1.369 (2)	C17—H17B	0.9800
N3—C19	1.401 (2)	C17—H17C	0.9800
N3—C16	1.471 (2)	C18—H18A	0.9800
N4—C15	1.363 (2)	C18—H18B	0.9800
N4—C21	1.399 (2)	C18—H18C	0.9800
N4—C23	1.474 (2)	C19—C21	1.344 (3)
C5—C7	1.519 (3)	C19—C20	1.491 (3)
C5—C6	1.528 (3)	C20—H20A	0.9800
C5—H5A	1.0000	C20—H20B	0.9800
C6—H6A	0.9800	C20—H20C	0.9800
C6—H6B	0.9800	C21—C22	1.494 (3)
C6—H6C	0.9800	C22—H22A	0.9800
C7—H7A	0.9800	C22—H22B	0.9800
C7—H7B	0.9800	C22—H22C	0.9800
C7—H7C	0.9800	C23—C25	1.515 (3)
C8—C10	1.342 (3)	C23—C24	1.527 (3)
C8—C9	1.502 (3)	C23—H23A	1.0000
C9—H9A	0.9800	C24—H24A	0.9800
C9—H9B	0.9800	C24—H24B	0.9800
C9—H9C	0.9800	C24—H24C	0.9800
C10—C11	1.494 (3)	C25—H25A	0.9800
C11—H11A	0.9800	C25—H25B	0.9800
C11—H11B	0.9800	C25—H25C	0.9800
			
C2—Fe1—C3	89.95 (9)	C12—C13—H13A	109.5
C2—Fe1—C1	120.54 (9)	C12—C13—H13B	109.5
C3—Fe1—C1	86.58 (8)	H13A—C13—H13B	109.5
C2—Fe1—C15	82.70 (8)	C12—C13—H13C	109.5
C3—Fe1—C15	172.20 (8)	H13A—C13—H13C	109.5
C1—Fe1—C15	99.38 (8)	H13B—C13—H13C	109.5
C2—Fe1—C4	131.97 (7)	C12—C14—H14A	109.5
C3—Fe1—C4	92.41 (7)	C12—C14—H14B	109.5
C1—Fe1—C4	107.49 (7)	H14A—C14—H14B	109.5
C15—Fe1—C4	90.60 (7)	C12—C14—H14C	109.5
C4—N1—C8	111.02 (15)	H14A—C14—H14C	109.5
C4—N1—C5	122.68 (14)	H14B—C14—H14C	109.5
C8—N1—C5	126.30 (16)	N4—C15—N3	103.82 (13)
C4—N2—C10	111.29 (16)	N4—C15—Fe1	128.43 (12)
C4—N2—C12	122.31 (15)	N3—C15—Fe1	127.75 (12)
C10—N2—C12	126.29 (16)	N3—C16—C18	113.85 (15)
C15—N3—C19	111.20 (14)	N3—C16—C17	109.91 (15)
C15—N3—C16	122.38 (14)	C18—C16—C17	112.75 (15)
C19—N3—C16	124.68 (14)	N3—C16—H16A	106.6
C15—N4—C21	111.40 (14)	C18—C16—H16A	106.6
C15—N4—C23	123.11 (14)	C17—C16—H16A	106.6
C21—N4—C23	125.32 (14)	C16—C17—H17A	109.5
O1—C1—Fe1	170.74 (17)	C16—C17—H17B	109.5
O2—C2—Fe1	175.73 (17)	H17A—C17—H17B	109.5
O3—C3—Fe1	175.51 (15)	C16—C17—H17C	109.5
N2—C4—N1	103.88 (14)	H17A—C17—H17C	109.5
N2—C4—Fe1	126.64 (12)	H17B—C17—H17C	109.5
N1—C4—Fe1	129.47 (12)	C16—C18—H18A	109.5
N1—C5—C7	114.16 (17)	C16—C18—H18B	109.5
N1—C5—C6	111.99 (17)	H18A—C18—H18B	109.5
C7—C5—C6	111.54 (16)	C16—C18—H18C	109.5
N1—C5—H5A	106.2	H18A—C18—H18C	109.5
C7—C5—H5A	106.2	H18B—C18—H18C	109.5
C6—C5—H5A	106.2	C21—C19—N3	106.66 (15)
C5—C6—H6A	109.5	C21—C19—C20	127.89 (17)
C5—C6—H6B	109.5	N3—C19—C20	125.29 (17)
H6A—C6—H6B	109.5	C19—C20—H20A	109.5
C5—C6—H6C	109.5	C19—C20—H20B	109.5
H6A—C6—H6C	109.5	H20A—C20—H20B	109.5
H6B—C6—H6C	109.5	C19—C20—H20C	109.5
C5—C7—H7A	109.5	H20A—C20—H20C	109.5
C5—C7—H7B	109.5	H20B—C20—H20C	109.5
H7A—C7—H7B	109.5	C19—C21—N4	106.83 (15)
C5—C7—H7C	109.5	C19—C21—C22	127.21 (18)
H7A—C7—H7C	109.5	N4—C21—C22	125.96 (17)
H7B—C7—H7C	109.5	C21—C22—H22A	109.5
C10—C8—N1	107.09 (16)	C21—C22—H22B	109.5
C10—C8—C9	128.0 (2)	H22A—C22—H22B	109.5
N1—C8—C9	124.8 (2)	C21—C22—H22C	109.5
C8—C9—H9A	109.5	H22A—C22—H22C	109.5
C8—C9—H9B	109.5	H22B—C22—H22C	109.5
H9A—C9—H9B	109.5	N4—C23—C25	111.40 (18)
C8—C9—H9C	109.5	N4—C23—C24	111.58 (18)
H9A—C9—H9C	109.5	C25—C23—C24	114.18 (19)
H9B—C9—H9C	109.5	N4—C23—H23A	106.4
C8—C10—N2	106.71 (17)	C25—C23—H23A	106.4
C8—C10—C11	128.2 (2)	C24—C23—H23A	106.4
N2—C10—C11	125.0 (2)	C23—C24—H24A	109.5
C10—C11—H11A	109.5	C23—C24—H24B	109.5
C10—C11—H11B	109.5	H24A—C24—H24B	109.5
H11A—C11—H11B	109.5	C23—C24—H24C	109.5
C10—C11—H11C	109.5	H24A—C24—H24C	109.5
H11A—C11—H11C	109.5	H24B—C24—H24C	109.5
H11B—C11—H11C	109.5	C23—C25—H25A	109.5
N2—C12—C13	113.80 (17)	C23—C25—H25B	109.5
N2—C12—C14	111.49 (16)	H25A—C25—H25B	109.5
C13—C12—C14	113.02 (16)	C23—C25—H25C	109.5
N2—C12—H12A	105.9	H25A—C25—H25C	109.5
C13—C12—H12A	105.9	H25B—C25—H25C	109.5
C14—C12—H12A	105.9		
			
C10—N2—C4—N1	−0.31 (19)	C10—N2—C12—C14	−70.2 (2)
C12—N2—C4—N1	−176.57 (15)	C21—N4—C15—N3	−3.00 (19)
C10—N2—C4—Fe1	178.52 (13)	C23—N4—C15—N3	172.56 (16)
C12—N2—C4—Fe1	2.3 (2)	C21—N4—C15—Fe1	176.66 (12)
C8—N1—C4—N2	0.41 (19)	C23—N4—C15—Fe1	−7.8 (2)
C5—N1—C4—N2	−179.58 (15)	C19—N3—C15—N4	2.90 (19)
C8—N1—C4—Fe1	−178.37 (13)	C16—N3—C15—N4	−162.70 (14)
C5—N1—C4—Fe1	1.6 (2)	C19—N3—C15—Fe1	−176.76 (12)
C2—Fe1—C4—N2	140.02 (15)	C16—N3—C15—Fe1	17.6 (2)
C3—Fe1—C4—N2	−127.88 (15)	C2—Fe1—C15—N4	88.48 (16)
C1—Fe1—C4—N2	−40.71 (16)	C1—Fe1—C15—N4	−31.39 (16)
C15—Fe1—C4—N2	59.32 (15)	C4—Fe1—C15—N4	−139.23 (15)
C2—Fe1—C4—N1	−41.4 (2)	C2—Fe1—C15—N3	−91.94 (15)
C3—Fe1—C4—N1	50.66 (16)	C1—Fe1—C15—N3	148.19 (15)
C1—Fe1—C4—N1	137.83 (15)	C4—Fe1—C15—N3	40.35 (15)
C15—Fe1—C4—N1	−122.14 (15)	C15—N3—C16—C18	−130.89 (17)
C4—N1—C5—C7	119.58 (19)	C19—N3—C16—C18	65.5 (2)
C8—N1—C5—C7	−60.4 (2)	C15—N3—C16—C17	101.53 (18)
C4—N1—C5—C6	−112.46 (19)	C19—N3—C16—C17	−62.1 (2)
C8—N1—C5—C6	67.5 (2)	C15—N3—C19—C21	−1.8 (2)
C4—N1—C8—C10	−0.4 (2)	C16—N3—C19—C21	163.43 (16)
C5—N1—C8—C10	179.62 (17)	C15—N3—C19—C20	173.86 (18)
C4—N1—C8—C9	−178.3 (2)	C16—N3—C19—C20	−20.9 (3)
C5—N1—C8—C9	1.7 (3)	N3—C19—C21—N4	−0.1 (2)
N1—C8—C10—N2	0.2 (2)	C20—C19—C21—N4	−175.63 (19)
C9—C8—C10—N2	178.0 (2)	N3—C19—C21—C22	180.0 (2)
N1—C8—C10—C11	−176.3 (2)	C20—C19—C21—C22	4.5 (3)
C9—C8—C10—C11	1.5 (4)	C15—N4—C21—C19	2.0 (2)
C4—N2—C10—C8	0.1 (2)	C23—N4—C21—C19	−173.42 (18)
C12—N2—C10—C8	176.17 (17)	C15—N4—C21—C22	−178.07 (19)
C4—N2—C10—C11	176.7 (2)	C23—N4—C21—C22	6.5 (3)
C12—N2—C10—C11	−7.2 (3)	C15—N4—C23—C25	111.8 (2)
C4—N2—C12—C13	−125.24 (19)	C21—N4—C23—C25	−73.2 (2)
C10—N2—C12—C13	59.1 (2)	C15—N4—C23—C24	−119.3 (2)
C4—N2—C12—C14	105.49 (19)	C21—N4—C23—C24	55.7 (3)

### Hydrogen-bond geometry (Å, º)

**Table:**

*D*—H···*A*	*D*—H	H···*A*	*D*···*A*	*D*—H···*A*
C25—H25*B*···O2^i^	0.98	2.42	3.154 (3)	131

Symmetry code: (i) −*x*+1, *y*+1/2, −*z*+1/2.
